# Local control after stereotactic radiation for thyroid cancer: a histology risk-stratified analysis

**DOI:** 10.3389/fonc.2026.1898567

**Published:** 2026-09-30

**Authors:** Bin Gui, Jayavignesh Sekar, Margaret Locke, Shyamali Saha, Mitchell Tulchinsky, Brianna Jones, Nagashree Seetharamu, Maged Ghaly

**Affiliations:** 1 Northwell Health, New Hyde Park, NY, United States; 2 Department of Radiation Medicine, New Hyde Park, NY, United States; 3 Department of Medical Oncology, New Hyde Park, NY, United States

**Keywords:** biological equivalent dose, histology -, metastatic thyroid cancer, SBRT (stereotactic body radiation therapy), thyroid cancer

## Abstract

**Purpose/Objective(s):**

Recurrent and metastatic non-anaplastic thyroid cancer has traditionally been considered relatively radioresistant. We evaluated whether anatomic site or histology risk group better predicts local control (LC) following stereotactic radiation therapy (SBRT/SRS).

**Materials/Methods:**

We retrospectively reviewed 32 patients with 66 treated lesions (42 skeletal, 24 non-skeletal) treated between 2012 and 2025. Histology was dichotomized into low-risk (papillary, follicular, medullary, Hürthle cell; n=61) and high-risk (poorly differentiated and squamous cell carcinoma; n=5). BED_10_ was calculated using α/β = 10 Gy. LC was defined per RECIST 1.1. Kaplan–Meier and log-rank tests were used. Intra-patient correlation was addressed via generalized estimating equations (GEE) and Cox shared frailty models. A multivariable GEE model included histology risk, site, BED_10_, PTV volume, prior surgery, and age.

**Results:**

Median follow-up was 15.0 months. Six local failures occurred (crude LC 90.9%); 1- and 2-year LC were 96.5% and 87.1%. Histology risk was the dominant determinant: low-risk histology achieved 95.1% crude LC versus 40.0% for high-risk (log-rank p=0.002). In multivariable GEE analysis, low-risk histology was independently associated with LC (OR 158.0, 95% CI 1.72–14,479.7; p=0.028), whereas skeletal site was not (OR 1.07, 95% CI 0.02–51.8; p=0.974). BED_10_ was not associated with LC after adjustment (p=0.752). Skeletal and non-skeletal LC were similar after histology stratification (95.0% vs 95.2% among low-risk). Treatment was well tolerated; no Grade ≥3 radiation toxicity, myelopathy, or fractures occurred.

**Conclusion:**

In stereotactic radiation for metastatic non-anaplastic thyroid cancer, histology risk grouping—not anatomic site or dose escalation—is the dominant predictor of local control. These findings support histology-based risk stratification for SRT patient selection.

## Introduction

Recurrent and metastatic non-anaplastic thyroid cancer has historically been regarded as relatively radioresistant, particularly when treated with conventional fractionation. However, the advent of stereotactic radiation therapy (SRT) has challenged this paradigm by enabling the delivery of highly conformal, ablative doses that may overcome intrinsic tumor resistance. Accumulating clinical experience indicates that high-dose hypofractionated radiation can achieve durable local control, especially in patients with oligometastatic or oligoprogressive disease (1–4).

In parallel, the therapeutic landscape of advanced thyroid cancer has evolved substantially with the incorporation of multikinase inhibitors (e.g., lenvatinib, cabozantinib), immune checkpoint inhibitors, and mutation-directed targeted therapies such as BRAF/MEK inhibition. While these systemic agents have improved progression-free survival, they frequently fail to control isolated progressing lesions, creating a clinical scenario in which local ablative therapies such as SRT play a critical role.

Despite growing evidence supporting SRT in thyroid cancer, most published series focus on single disease sites, particularly spine metastases, with limited data comparing outcomes across different anatomical locations. Furthermore, the relationship between radiation dose, biologically effective dose (BED_10_), tumor biology, and treatment context remains incompletely defined (5–8). Whether skeletal and non-skeletal metastases behave differently in response to SRT has not been systematically evaluated.

Poorly differentiated thyroid carcinoma (PDTC) and squamous cell carcinoma of thyroid origin represent biologically aggressive subtypes with distinct clinical behavior and worse prognosis compared with well-differentiated papillary, follicular, and medullary carcinomas (9–14). Whether these high-risk histologies respond differently to ablative radiation doses—and whether such differences confound apparent site-specific outcomes—has not been examined. We therefore performed a comprehensive analysis of patients with recurrent and metastatic non-anaplastic thyroid cancer treated with SRT, with specific emphasis on histology risk stratification, site-specific outcomes, detailed dosimetric parameters, and interaction with systemic therapy.

## Materials and methods

### Patient selection

This retrospective single-institution study evaluated outcomes of stereotactic radiation therapy for patients with recurrent or metastatic non-anaplastic thyroid carcinoma treated between 2012 and 2025. A total of 32 patients with 66 treated lesions were included, with 18 male and 14 female patients. Clinical, treatment, dosimetric, and follow-up data were collected under an approved institutional review board protocol. Lesions were categorized into skeletal (n=42, 63.6%) and non-skeletal (n=24, 36.4%) sites. Histologies included papillary (n=36 lesions), follicular (n=17), medullary (n=7), poorly differentiated (n=3), squamous cell (n=2), and Hürthle cell carcinoma (n=1). Squamous cell carcinoma cases were confirmed as thyroid primary origin through immunohistochemistry (thyroid transcription factor-1 positive, PAX8 positive) and exclusion of head-and-neck primary by dedicated otolaryngologic examination and PET-CT imaging. Poorly differentiated carcinoma was distinguished from anaplastic thyroid carcinoma by retained follicular architecture, absence of widespread necrosis, and TP53 mutation status where available.

### Histology risk stratification

Based on established differences in radiosensitivity and clinical behavior, we grouped histologies into 2 category. High-risk histology comprised poorly differentiated thyroid carcinoma and squamous cell carcinoma. Low-risk histology comprised papillary, follicular, medullary, and Hürthle cell carcinoma. This grouping was defined before statistical analysis.

### Treatment

Stereotactic radiation was delivered using SRS or SBRT techniques. Prescription regimens included 16–18 Gy ×1, 24–27 Gy in 3 fractions, and 35–45 Gy in 5 fractions, selected based on lesion location, prior treatment, and proximity to organs at risk. Skeletal lesions were preferentially treated with single or 3-fraction regimens, whereas non-skeletal lesions more commonly received 5-fraction SBRT.

### Dosimetry

Dosimetric parameters were available for 61 lesions. The median PTV was 101.1 cc (range 4.2–1117.2 cc). Plan quality was consistently high, with median D90 = 100.7%, median MPD/D50 = 1.048, and volumetric parameters of V100 = 96.8 cc, V95 = 110.0 cc, V90 = 122.7 cc. The median conformity index was 0.97.

### Dose calculation

Biologically effective dose (BED_10_) was calculated using α/β = 10 Gy, consistent with convention for epithelial tumors and SBRT dose–response modeling. We acknowledge that the α/β ratio for differentiated thyroid cancer is not well established and may be lower; therefore, BED_10_ should be interpreted as a standardized approximation rather than a precise biologic measure. Skeletal lesions received lower doses (median BED_10_ 50.4 Gy, range 41.6–72.0 Gy) compared with non-skeletal lesions (median 72.0 Gy, range 35.7–100.0 Gy; p<0.001), reflecting technical limitations and treatment selection.

### Follow-up and outcomes

Patients were followed according to consensus practice, with evaluation at 1 month post-SRT, every 3 months for the first year, and annually thereafter, unless clinically indicated. Local control (LC) was defined as absence of radiographic progression within the treated lesion based on follow-up imaging per RECIST 1.1 criteria. Time to local failure was calculated from the date of radiation therapy to the date of first documented progression or last imaging. Kaplan–Meier methodology was used to estimate local control over time, and comparisons between subgroups were performed using log-rank testing. Median follow-up was calculated using the reverse Kaplan–Meier method. Comparisons between groups were performed using the Wilcoxon rank-sum test for continuous variables and χ² or Fisher’s exact test for categorical variables, as appropriate.

### Statistical analysis

Because multiple lesions from the same patient were included (66 lesions from 32 patients), standard Kaplan–Meier and log-rank methods—which assume independence—were supplemented with analyses that account for intra-patient correlation. The primary analysis was a multivariable generalized estimating equation (GEE) logistic regression model for local control, clustered by patient ID using an exchangeable working correlation structure. Covariates included histology risk (low vs high), skeletal site (yes vs no), BED_10_ (continuous), PTV volume (continuous), prior thyroidectomy (yes vs no), and age (continuous). Sensitivity analyses included Cox proportional hazards models with shared gamma frailty (random effect at the patient level) and robust sandwich variance estimators clustered by patient. All analyses were performed in SAS 9.4 (SAS Institute, Cary, NC). Statistical significance was defined as a two-sided p-value <0.05.

## Results

### Cohort and treatment characteristics

A total of 66 lesions from 32 patients were analyzed (**Table 1**). Median age was 70 years (range 26–84). By risk grouping, 61 lesions (92.4%) were low-risk and 5 lesions (7.6%) were high-risk. Skeletal lesions accounted for 42 lesions (63.6%), comprising 23 non-spine bone and 19 spine lesions. Non-skeletal lesions comprised 23 head-and-neck and 1 soft-tissue lesion. Prior thyroidectomy was recorded in 20 lesions (30.3%) and prior radioactive iodine (RAI) therapy in 18 lesions (27.3%). Distant metastasis at diagnosis was documented in 33 lesions (50.0%).

**Table 1:** Patient and lesion characteristics.

Characteristic	n (%) or Median (range)
Patients	32
Male	18 (56.2%)
Female	14 (43.8%)
Age, years	70 (26–84)
Lesions	66
Lesions per patient, median (IQR)	2 (1–2)
Histology (lesion-level)	
Papillary carcinoma	36 (54.5%)
Follicular carcinoma	17 (25.8%)
Medullary carcinoma	7 (10.6%)
Poorly differentiated thyroid carcinoma	3 (4.5%)
Squamous cell carcinoma	2 (3.0%)
Hürthle cell carcinoma	1 (1.5%)
Histology risk group	
Low-risk	61 (92.4%)
High-risk	5 (7.6%)
Anatomic site	
Skeletal	42 (63.6%)
Non-spine bone	23 (34.8%)
Spine	19 (28.8%)
Non-skeletal	24 (36.4%)
Head and neck	23 (34.8%)
Soft tissue	1 (1.5%)
Prior thyroidectomy	20 (30.3%)
Prior RAI therapy	18 (27.3%)
Distant metastasis at diagnosis	33 (50.0%)
PTV volume, cc	101.1 (4.2–1117.2)
BED_10_, Gy	51.3 (35.7–100.0)
Skeletal	50.4 (41.6–72.0)
Non-skeletal	72.0 (35.7–100.0)
Fractionation	
1-fraction	22 (33.3%)
3-fraction	18 (27.3%)
5-fraction	26 (39.4%)
Dosimetric parameters (n=61)	
D90, %	100.7 (0.6–100.7)
V95, cc	110.0 (10.8–1140.8)
Conformity index	0.97 (0.49–1.43)

Median BED_10_ was 51.3 Gy (range 35.7–100.0). Fractionation was 1-fraction in 22 lesions (33.3%), 3-fraction in 18 (27.3%), and 5-fraction in 26 (39.4%). Dosimetric quality was high across all lesions (median D90 100.7%, median CI 0.97).

### Local control outcomes

Six local failures were observed, yielding a crude LC of 90.9%. One- and 2-year LC for the entire cohort were 96.5% and 87.1%, respectively.

### Histology risk

Histology risk was the dominant determinant of outcome. Low-risk histology demonstrated 95.1% crude LC (58/61) versus 40.0% (2/5) for high-risk histology. Kaplan–Meier 1-year LC was 100% for low-risk versus 60.0% for high-risk; 2-year LC was 92.1% versus 40.0% (log-rank p=0.002). All six local failures occurred in high-risk histology or in low-risk histology with concurrent high-risk features.

### Anatomic site

Skeletal lesions achieved 92.9% crude LC (39/42) versus 87.5% (21/24) for non-skeletal lesions. One-year LC was 97.5% versus 94.7%; 2-year LC was 90.0% versus 82.1% (log-rank p=0.42). The difference was not statistically significant. After stratifying by histology risk, the site difference was negligible: among low-risk histologies, skeletal LC was 95.0% (38/40) and non-skeletal LC was 95.2% (20/21) (**Figure 1**).

**Figure 1:**
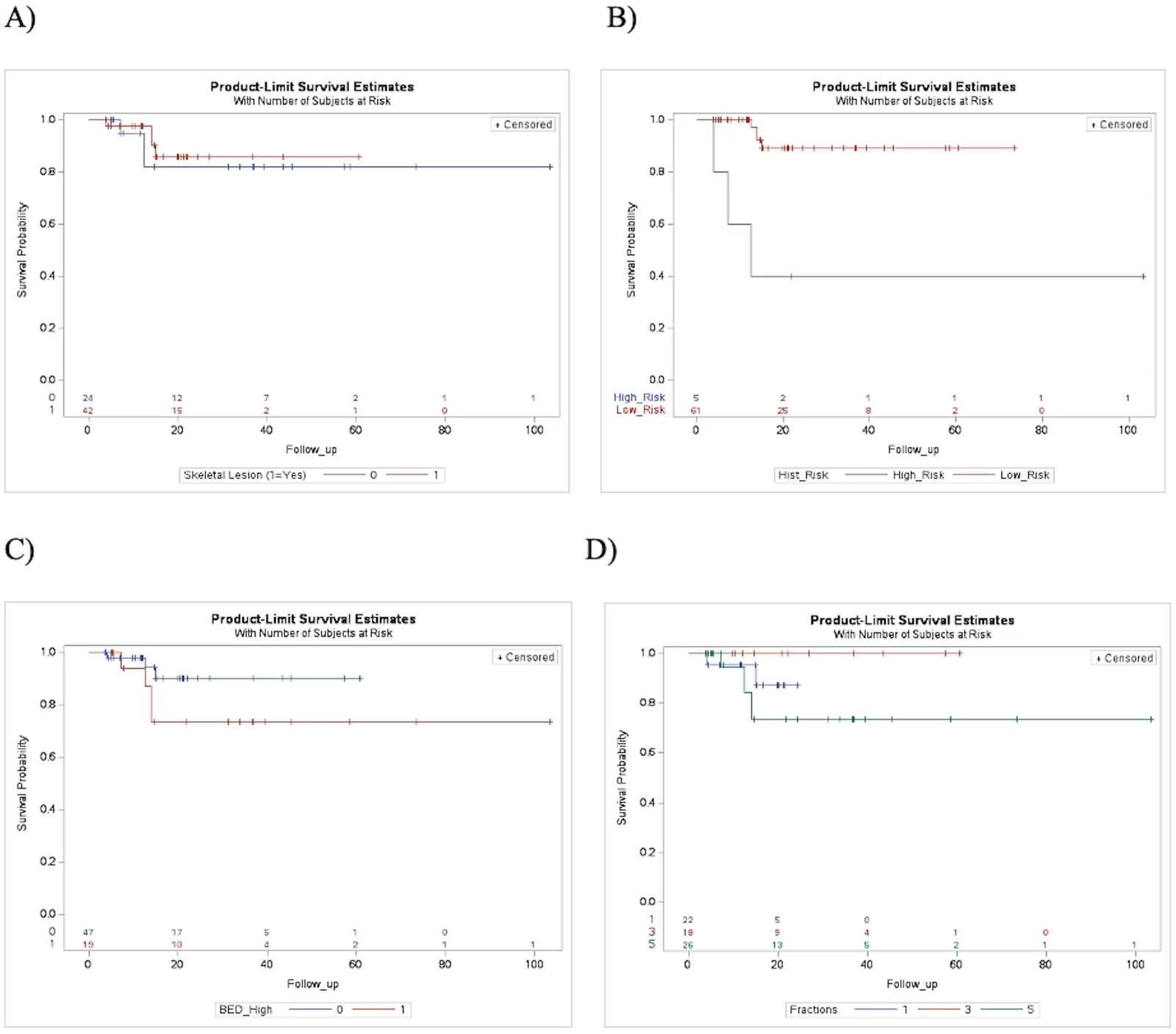
(**A**) Local control by anatomic site. Skeletal lesions demonstrated numerically higher but statistically similar local control compared with non-skeletal lesions (log-rank p=0.42). **(B)** Local control by histology risk group. Low-risk histology (papillary, follicular, medullary, Hürthle cell) achieved significantly superior local control compared with high-risk histology (poorly differentiated and squamous cell carcinoma) (log-rank p=0.002). **(C)** Local control by biologically effective dose (BED_10_). Lesions receiving BED_10_ ≥60 Gy (BED_high=1) showed lower crude local control (78.9%) compared with those receiving <60 Gy (95.7%, log-rank p=0.07); however, this inverse relationship was entirely attributable to confounding, as higher BED was selectively delivered to high-risk, non-skeletal lesions. After multivariable adjustment, BED_10_ was not independently associated with local control. **(D)** Local control by fractionation regimen. Single-fraction, 3-fraction, and 5-fraction regimens achieved crude local control of 95.5%, 100%, and 80.8%, respectively (log-rank p=0.09). The lower control in the 5-fraction group was driven by inclusion of high-risk histologies and larger-volume non-skeletal lesions; after adjustment, fractionation was not independently predictive of local control. Numbers below the x-axis indicate patients at risk at each time point. *p < 0.05.

To address differential censoring between skeletal and non-skeletal lesions, we performed a sensitivity analysis restricted to lesions with ≥6 months of follow-up (n=53 of 66, 80.3%). In this restricted cohort, overall LC was 90.6%, low-risk histology LC was 93.9%, and high-risk histology LC was 50.0%—all consistent with the primary analysis. The skeletal versus non-skeletal difference persisted (94.1% vs 84.2%) but remained confounded by histology risk distribution.

### Multivariable analysis

In the primary GEE logistic model (**Table 2**), low-risk histology was strongly associated with LC (adjusted OR 158.0, 95% CI 1.72–14,479.7; p=0.028). Skeletal site was not associated with LC (aOR 1.07, 95% CI 0.02–51.8; p=0.974). BED_10_ showed no association (aOR 0.98 per Gy, 95% CI 0.86–1.12; p=0.752). Older age was associated with better LC (aOR 1.13 per year, 95% CI 1.03–1.24; p=0.007), likely reflecting selection bias toward SRT in older patients with indolent disease. PTV volume (p=0.286) and prior surgery (p=0.868) were not significant (**Figure 2**).

**Table 2:** Multivariable GEE logistic regression for local control (clustered by patient).

Variable	Adjusted OR	95% CI	p-value
Low-risk histology (ref: high-risk)	158.0	1.72–14,479.7	0.028
Skeletal site (ref: non-skeletal)	1.07	0.02–51.8	0.974
BED_10_ (per Gy)	0.98	0.86–1.12	0.752
PTV volume (per cc)	1.00	0.99–1.00	0.286
Prior thyroidectomy (ref: no)	0.78	0.04–13.9	0.868
Age (per year)	1.13	1.03–1.24	0.007

**Figure 2:**
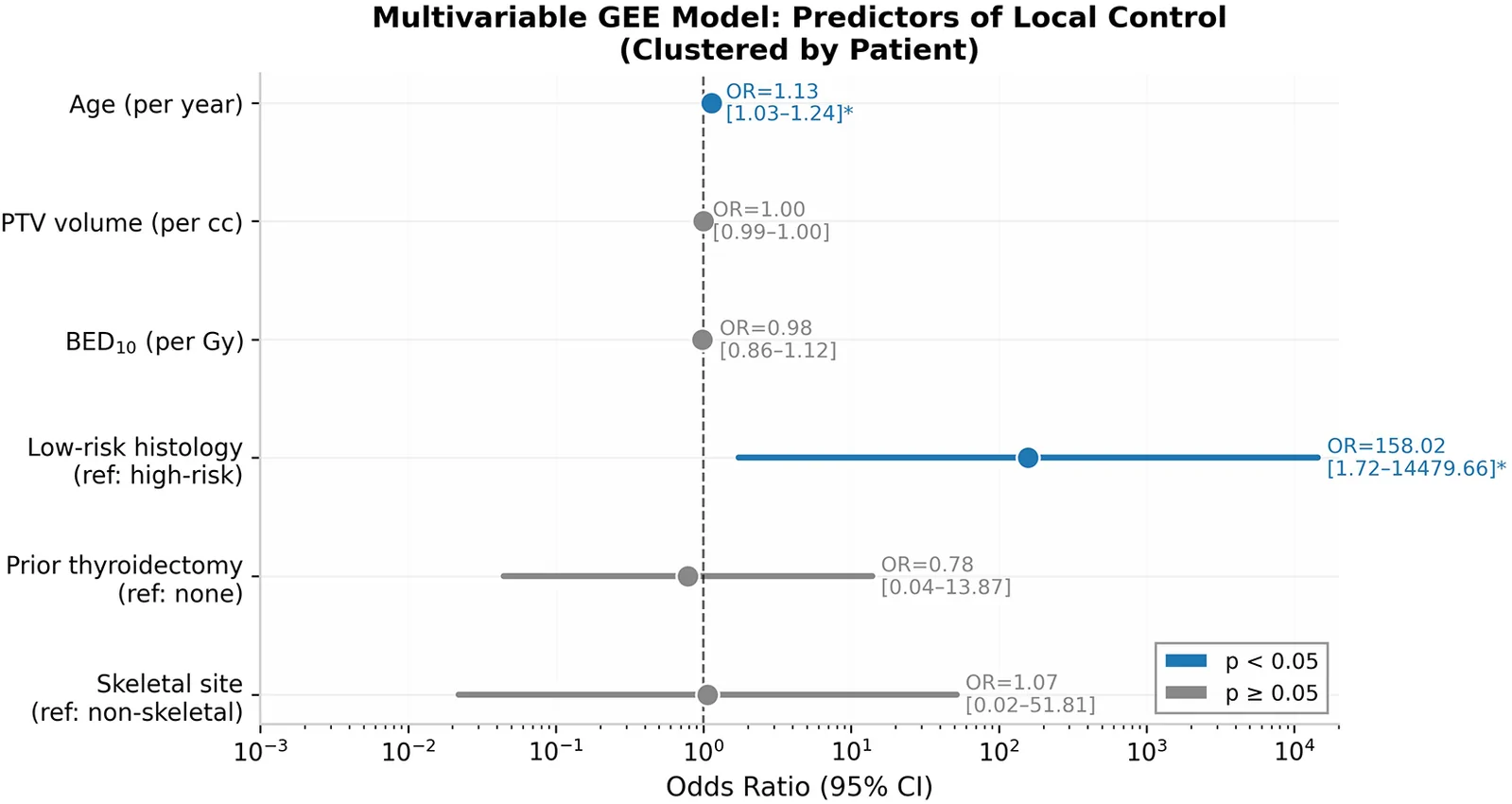
Multivariable generalized estimating equation (GEE) logistic regression for local control, clustered by patient with exchangeable working correlation. Low-risk histology and older age were significantly associated with improved local control. Skeletal site, BED_10_, PTV volume, and prior thyroidectomy were not independently predictive. Error bars represent 95% confidence intervals. The vertical dashed line indicates an odds ratio of 1.0. *p < 0.05.

Results were consistent in the Cox shared frailty model: low-risk histology HR 0.12 (95% CI 0.02–0.71; p=0.019), skeletal site HR 0.85 (95% CI 0.18–3.98; p=0.84).

### Dose and fractionation

BED_10_ ≥60 Gy was associated with lower crude LC (78.9% vs 95.7% for <60 Gy), but this was entirely explained by confounding: high-BED lesions were disproportionately non-skeletal and high-risk histology. After multivariable adjustment, BED was not predictive of LC (**Figure 3**).

**Figure 3:**
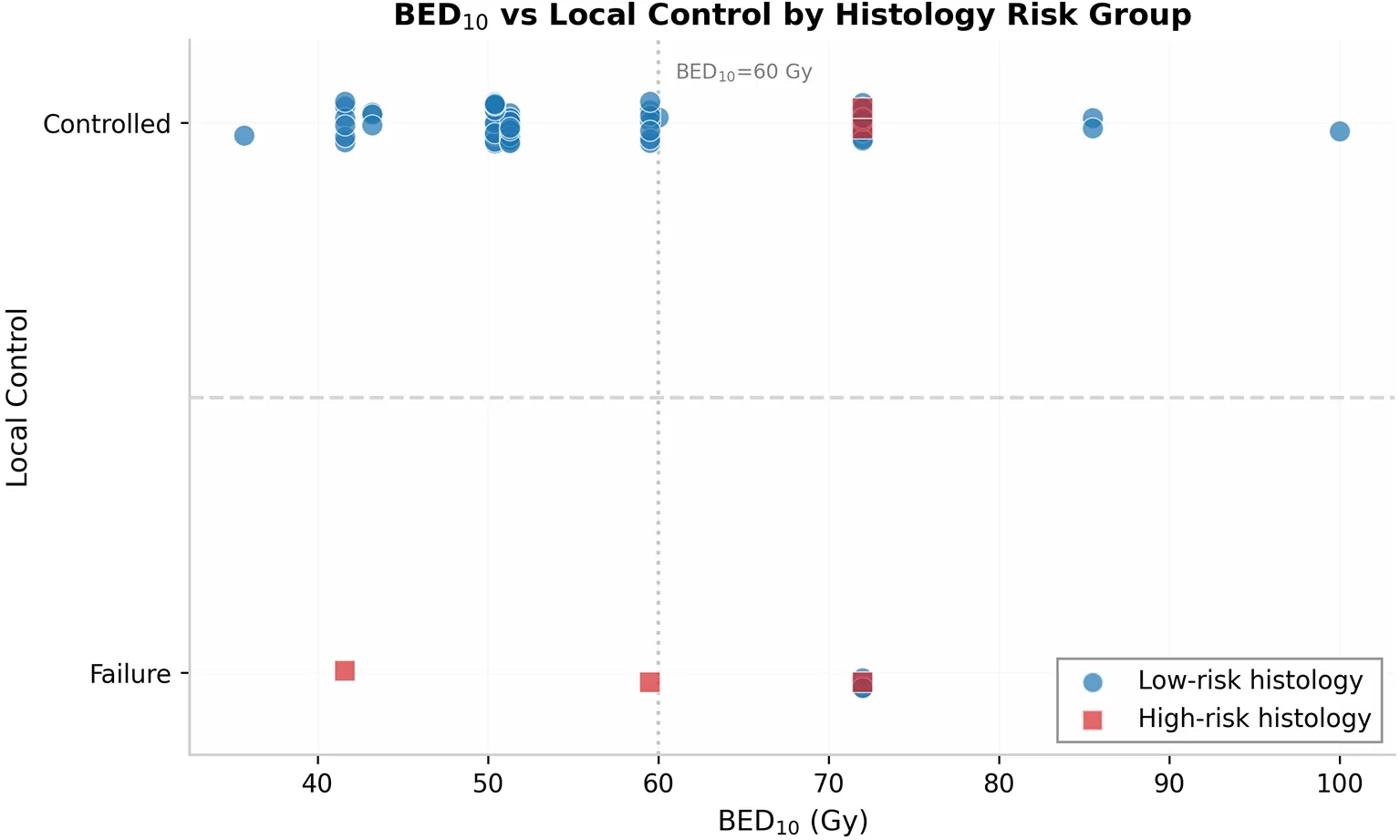
Scatter plot of biologically effective dose (BED_10_) versus local control, stratified by histology risk group. Each point represents an individual lesion. The dashed horizontal line separates controlled from failed lesions. The dotted vertical line indicates BED_10_ = 60 Gy. High-risk histology lesions (squares) cluster among failures regardless of BED_10_, whereas low-risk histology lesions (circles) achieve control across the full dose range. The apparent inverse relationship between BED and local control is explained by confounding: higher BED was selectively delivered to high-risk, non-skeletal lesions.

Single-fraction, 3-fraction, and 5-fraction regimens achieved crude LC of 95.5%, 100%, and 80.8%, respectively. The lower LC in the 5-fraction group was driven by inclusion of high-risk histologies and larger-volume non-skeletal lesions; after adjustment, fractionation was not independently associated with LC.

### Toxicity

Treatment was well tolerated. Acute radiotherapy-related toxicities included Grade 1 hoarseness in 3 patients (9.4%), Grade 2 dysphagia in 1 patient (3.1%), Grade 1 dysphagia in 1 patient (3.1%), and Grade 1 dry skin in 1 patient (3.1%). Toxicities were graded per CTCAE v5.0. No Grade ≥3 acute or late toxicities, radiation myelopathy, or treatment-related fractures were observed. With median follow-up of 15.0 months, late toxicity data remain immature.

## Discussion

Stereotactic radiation therapy achieves high local control in metastatic non-anaplastic thyroid cancer, with 1- and 2-year LC rates of 96.5% and 87.1% in this cohort. The present study demonstrated that local control is strongly associated with the histology risk grouping rather than anatomic site or dose escalation.

Our initial univariate comparison suggested a modest numerical advantage for skeletal lesions (92.9% vs 87.5% crude LC). However, this difference was not statistically significant and disappeared after accounting for histology risk. Among low-risk histologies, skeletal and non-skeletal sites achieved nearly identical control (95.0% vs 95.2%). The apparent site effect was confounded by the distribution of high-risk histologies: poorly differentiated and squamous carcinomas were overrepresented in non-skeletal sites and received higher BED, creating a spurious inverse dose–response relationship. Once histology was included in the model, neither site nor BED remained predictive.

This finding has important clinical implications. Prior literature has emphasized spine-specific or bone-specific SBRT outcomes in thyroid cancer, often reporting excellent LC in osseous metastases. These reports may have been enriched for well-differentiated histologies. Conversely, the inferior outcomes reported in soft-tissue and nodal sites may reflect referral patterns that concentrate aggressive histologies in these locations. A histology-first, rather than site-first, approach to prognostication appears warranted.

The strong performance of low-risk histologi es (95% crude LC) supports aggressive local ablation in differentiated thyroid cancer, even at lower BED. The dismal outcomes in high-risk histology (40% crude LC) suggest that SBRT alone may be insufficient for PDTC and squamous variants, and that these patients may require intensified systemic therapy or alternative local strategies.

The inverse relationship between BED_10_ and LC observed in univariate analysis (78.9% LC for BED ≥60 Gy vs 95.7% for <60 Gy) may be attributable to treatment selection bias. Higher BED was preferentially delivered to non-skeletal, gross, and biologically aggressive lesions, which inherently carry elevated failure risk. After multivariable adjustment including histology risk, BED maintained a neutral association with outcome (aOR 0.98 per Gy, p=0.752), supporting conventional radiobiologic expectations and refuting any true paradox.

We acknowledge several limitations. The sample size is modest, particularly for high-risk histology (n=5 lesions). The retrospective design introduces selection bias in dose assignment and patient selection. Follow-up was shorter and more variable for non-skeletal lesions (median 7.2 vs 20.5 months), though with only six total events, the impact of differential censoring on estimates is limited. A sensitivity analysis restricted to lesions with ≥6 months of follow-up yielded consistent results. Dosimetric parameters were unavailable for 5 lesions. Systemic therapy and molecular data were incompletely recorded, precluding formal analysis of treatment interaction. The claims regarding biological mechanisms underlying the histology effect remain speculative in the absence of translational correlatives. Finally, the GEE model exhibited quasi-complete separation due to the small high-risk subgroup (n = 5 lesions); consequently, the odds ratio point estimate should be interpreted with caution, and the confidence interval is provided to reflect uncertainty rather than precision. The consistent direction of effect in the Cox shared frailty model (HR 0.12, 95% CI 0.02–0.71; p = 0.019) supports the robustness of the histology association.

## Conclusion

Stereotactic radiation therapy achieves excellent local control in low-risk histology metastatic thyroid cancer, irrespective of anatomic site. High-risk histology (poorly differentiated and squamous cell carcinoma) is the dominant predictor of local failure. Skeletal site conferred no independent advantage after adjustment for histology. These findings support histology-based risk stratification for patient selection and counseling when considering SRT in advanced thyroid cancer.

## Data Availability

The raw data supporting the conclusions of this article will be made available by the authors, without undue reservation.
